# Evaluation of protein secondary structure from FTIR spectra improved after partial deuteration

**DOI:** 10.1007/s00249-021-01502-y

**Published:** 2021-02-03

**Authors:** Joëlle De Meutter, Erik Goormaghtigh

**Affiliations:** Center for Structural Biology and Bioinformatics, Laboratory for the Structure and Function of Biological Membranes, Campus Plaine CP206/02, Université Libre de Bruxelles, B1050 Brussels, Belgium

**Keywords:** FTIR spectroscopy, Secondary structure, Protein spectroscopy, Protein microarrays, Hydrogen deuterium exchange

## Abstract

FTIR spectroscopy has become a major tool to determine protein secondary structure. One of the identified obstacle for reaching better predictions is the strong overlap of bands assigned to different secondary structures. Yet, while for instance disordered structures and α-helical structures absorb almost at the same wavenumber, the absorbance bands are differentially shifted upon deuteration, in part because exchange is much faster for disordered structures. We recorded the FTIR spectra of 85 proteins at different stages of hydrogen/deuterium exchange process using protein microarrays and infrared imaging for high throughput measurements. Several methods were used to relate spectral shape to secondary structure content. While in absolute terms, β-sheet is always better predicted than α-helix content, results consistently indicate an improvement of secondary structure predictions essentially for the α-helix and the category called “Others” (grouping random, turns, bends, etc.) after 15 min of exchange. On the contrary, the β-sheet fraction is better predicted in non-deuterated conditions. Using partial least square regression, the error of prediction for the α-helix content is reduced after 15-min deuteration. Further deuteration degrades the prediction. Error on the prediction for the “Others” structures also decreases after 15-min deuteration. Cross-validation or a single 25-protein test set result in the same overall conclusions.

**Supplementary Information:**

The online version contains supplementary material available at 10.1007/s00249-021-01502-y.

## Introduction

Proteins are widely used as therapeutics in the biopharmaceutical industry and in food industry (Dimitrov 2012). Their characterisation is an essential step in the development and quality control processes (Raynal et al. 2014; Rogstad et al. 2019). Quality is indeed an essential parameter for drug approval by the FDA and other similar agencies. Proteins are prone to structural modification during production, storage and transport (shaking). Protein characterization is made arduous by their complexity, size and unstable 3D structure. This highlights the importance of monitoring and quickly obtaining information on the structure of a protein set. Fourier transform infrared spectroscopy (FTIR) is a powerful tool to assess protein secondary structure (Byler and Susi 1986; Susi and Byler 1986a; Surewicz and Mantsch 1988; Goormaghtigh et al. 1990, 2006, 2009; Lee et al. 1990; Prestrelski et al. 1992; Arrondo et al. 1993; Pribic et al. 1993; Oberg et al. 2003, 2004; Hering et al. 2004; Navea et al. 2005, 2006; Barth 2007; Wang et al. 2008; Wilcox et al. 2016) and simultaneously protein glycan content (Derenne et al. 2020). Recently, we proposed a new approach for evaluating protein secondary structure in a high throughput way, combining infrared imaging and protein microarray printing (De Meutter and Goormaghtigh 2021) . We also designed a protein library, made out of 92 soluble proteins (cSP92) carefully selected for spectroscopic calibration and commercially available to everyone (De Meutter and Goormaghtigh 2020b). The protein library is well characterized and we have shown how the structures present in the set cover the space of secondary structures and folds described by CATH classification (Orengo et al. 1997). Moreover, we also showed how the relatively small set of proteins present in cSP92 presents similar distribution of structural features as the overall protein set described in the PDB. The FTIR spectra obtained from the 92 calibration set proteins allowed building prediction models of protein secondary structure (De Meutter and Goormaghtigh 2021) based on high performance algorithms used in Chemometrics such as Partial least square (PLS) (Geladi and Kowalski 1986; Wold et al. 2001), Support vector machine (SVM) (Tange et al. 2015; Ghorbani et al. 2016) or Ascending stepwise linear regression (ASLR) (Goormaghtigh et al. 2006, 2009). One of the identified potential obstacles for reaching better predictions is the strong overlap of the bands assigned to different secondary structure types in the amide vibration region of the spectrum. It is well known that amide I (about 80% C = O stretching) and amide II (mainly δ(N–H and ν(C-N)) bands are the most useful for secondary structure determination (Susi 1972). In the amide I band, the disordered structures and α-helical structures absorb almost at the same wavenumber (Byler and Susi 1986; Susi and Byler 1986b; Goormaghtigh et al. 1990), reviewed in (Goormaghtigh et al. 1994a). A potential way to improve accuracy of secondary structure evaluation would be to use exchange of hydrogen atoms of the protein by deuterium (HDX) to better separate the absorption bands (Zuber et al. 1992; Iloro et al. 2008). Indeed, in amide I band, HDX induces a slight of 5–10 cm^−1^ to lower wavenumbers upon N–H deuteration (Susi 1972), reviewed in (Goormaghtigh et al. 1994a), while the amide II disappears and a new band, called amide II’, appears about 100 cm^−1^ below. It must be noted that the smaller shifts reported in the literature (< 10 cm^−1^) are likely to be due to incomplete deuteration as, when proteins are fully deuterated, e.g. obtained from cells growing in ^2^H_2_O (Sivakumar et al. 2005), the shift is around 12 cm^−1^. A key factor is that exchange is expected to proceed much more rapidly for peptidic elements that are more exposed and/or less stable, as the unordered fraction is expected to be. For structured polypeptide chains, exchange is indeed slow and rates are determined by the small fraction of the time that the protein experiences in a transiently unfolded conformation (Hvidt and Nielsen 1966; Englander and Kallenbach 1983a; Englander et al. 2003; Zhang 2020). The displacement of the Amide I band of the unordered fraction to lower wavenumbers should therefore initially result in a better separation from the band assigned to α-helices. Baello et al. already showed improved prediction of protein secondary structure on a set of 19 proteins in solution after partial HD exchange (Baello et al. 2000).

While measuring spectra of proteins at different stage of HD exchange is tedious, it becomes particularly convenient upon combining microarray printing and infrared imaging (De Meutter et al. 2016, 2017). Indeed, a cell formed by the microarray-bearing BaF_2_ slide and a clean BaF_2_ slide separate from the first one by a spacer can be flushed by ^2^H_2_O-saturated N_2_ flow. Exchange occurs, then simultaneously for all proteins and single images covering ca 96 spots of proteins can be recorded on-line at selected time points. The advantage of this method is that it allows the simultaneous recording of all the spectra of the proteins printed on a microarray, at any HD exchange time.

This work highlights an improvement of secondary structure predictions essentially for the α-helix and the category called “Others” (grouping random, turns, bends, etc.) in partial HDX conditions. On the contrary, β-sheet fraction is better predicted in non-deuterated conditions.

## Materials and methods

### Proteins

The proteins used in this work are a subset of the cSP92 protein library. The list of the proteins has been published with their commercial source and their characterization (sequence, purity,…) (De Meutter and Goormaghtigh 2020b). Seven proteins of cSP92 were discarded due to the poor quality of the spectra. This low quality was the result of low-protein concentration related to the small quantities available, of the passage of the IR beam through 2 BaF_2_ windows and of the use of the 4 × objective required to observe simultaneously 96 protein spots (see below). Table S1 reports the subset of 85 proteins selected. Protein samples were solubilized at a final concentration of 10–20 mg/ml in 4 mM Hepes, 85 mM NaCl. Buffer solutions were filtered on 0.2 μm filters before use. To avoid contributions of the original buffer, salts and/or additives of preparation or purification, samples were de-salted and buffers exchanged against 4 mM Hepes, 85 mM NaCl (5%), pH between 7.4 and 7.6 except for a few proteins as described in (De Meutter and Goormaghtigh 2020b). Buffer exchange of ca 100 µl sample was achieved through filtration by 5 repetitive cycles (Amicon Ultra-0.5 ml Centrifugal Filters 3 K). Around 75 µl were collected. Alternatively, they were passed twice through size exclusion centrifuge mini column (Bio-Rad Micro Bio-Spin 3kD) equilibrated with buffer. Purity and integrity of the acquired proteins were then controlled by SDS Page (4–20% Mini-PROTEAN Precast Protein Gels, Bio-Rad), all protein used were found to have a purity larger than 85%.

### Protein microarrays printing

The experimental procedure is described in detail elsewhere (De Meutter et al. 2016, 2017); 100 pl protein drops (proteins dissolved at 5–10 mg ml^−1^ in 2 mM Hepes buffer pH 7.0/ethylene glycol 1/1 v/v) have been printed with an Arrayjet Marathon noncontact inkjet Microarrayer (ArrayJet, Roslin, UK) forming microarrays on BaF_2_ surfaces. Spot diameter was about 80 μm. Spot-to-spot distances in the X and Y directions were 200 μm, resulting in ca 2,000 protein samples per cm^2^. Before use, the protein microarrays were dried in a dessicator under vacuum. All proteins were recorded in quadruplicates obtained from the same batch of protein.

### FTIR imaging of protein microarrays

FTIR imaging of protein microarrays has been described earlier (De Meutter et al. 2016, 2017). Spectra were recorded as the average of 64 scans per pixel, between 3650 and 900 cm^−1^ at a nominal resolution of 8 cm^−1^. FTIR data were collected in transmission mode using an Agilent mid-IR imager equipped with a liquid nitrogen cooled 128 × 128 Mercury Cadmium Telluride (MCT) Focal Plane Array (FPA) detector and a 4 × objective.

Automated spectrum extraction has already been described (De Meutter et al. 2017), including the procedure followed to subtract the background. With the 4 × objective, a single protein spot usually contained ca 20 pixels, i.e. 20 spectra. After correction for background, spectra filtered for signal-to-noise ratio and maximum absorbance were averaged. Finally, the average spectra of quadruplicates obtained for a same protein were averaged, yielding one spectrum per protein. Spectra were then baseline-corrected by subtraction of a straight line interpolated between the spectral points at 1720 and 1480 cm^−1^. Scaling was obtained by vector normalization between 1720 and 1590 cm^−1^. Precise peak position (Figure S2) was obtained by fitting 11 data points before and after the approximate maximum by a third-order polynomial and finding the roots of its derivative as described (Derenne et al. 2013).

### Hydrogen deuterium exchange

A homemade sealed cup was designed specifically for this experimentation. The assembly consisted of two BaF_2_ slides used in turn to form a vessel, the bottom one supported the printed microarray and the top one closed the cell. In between, along the edges of the slides, UHU^®^ Patafix adhesive paste (Bolton Adhesives) was used to hermetically seal the two surfaces and two pieces of catheter ensured the entry and exit of ^2^H_2_O-saturated nitrogen flow. A N_2_ flow bubbled in 3 vials containing ^2^H_2_O placed in series before being sent to the sample at a flow rate of 100 ml/min. Once vessel and catheters were set up and positioned under the microscope, the focus was adjusted and the device remained in place for the whole duration of the exchange experiment (about 24 h). The use of a 4 × objective allowed recording an entire microarray in one single image. A microarray with 96 spots covered an area of about 3.75 mm^2^. The area of a 4 × magnification infrared image covers 6.9 mm^2^. IR images of the microarrays were recorded at 4 deuteration times: t_0_, no deuteration; t_15_: 15′25″ ± 1′; t_105_:1h 45′ ± 12′ and t_24h_ 24 h ± 1 h the day after.

### Secondary structures

In this work, secondary structure content are reported in % of the total amino acid content. The identities of the high-resolution PDB files obtained from the PDB repository was previously reported along with the secondary structure content obtained by applying the DSSP algorithm (De Meutter and Goormaghtigh 2020b). According to DSSP nomenclature, α-helix is symbolized by the letter H and β-sheet by E (Kabsch and Sander 1983). The “minor” structures such as 3_10_-helix (G), π-helix (I), helix-turn (T), beta bridge (B) and bend (S) defined by DSSP could not be predicted with sufficient accuracy (De Meutter and Goormaghtigh 2021) and will not be investigated independently here. We therefore define a category called “Others” computed as 100-H-E. As previously proposed by (Kalnin et al. 1990), the α-helix structure was split into “ordered” and “disordered” helix. The “ordered” helix content was obtained after amputation of two amino acids residues at both ends of the α-helices. The tips of the α-helices not included in the previous group are assigned to “disordered” helix fraction. It was originally reported that parallel and antiparallel β-sheet cannot be differentiated (Susi and Byler 1987) but it has also been shown in a number of cases that parallel and antiparallel β-sheets have distinct FTIR spectra (Cerf et al. 2009; Celej et al. 2012). The β-sheet structure was therefore split into parallel and antiparallel β-sheet, respectively. It was found (data not shown) that the parallel β-sheet and ordered α-helix contents could not be predicted satisfactorily because of too low variance in the protein set. Results will not be further discussed here. On the contrary, the α-helix, ordered α-helix, β-sheet, antiparallel β-sheet, and “Others” structure contents present enough variance in the protein set to generate good prediction models. Content in ordered/disordered helix and parallel/antiparallel β-sheet were compiled from DSSP files. All structural features have been extracted and tabulated from the DSSP files by a module of the home-made Kinetics software running under Matlab, as described in (De Meutter and Goormaghtigh 2020b).

### Chemometrics

Partial least square regression (PLS) adequately addresses the problem of co-linearity in multivariate linear regressions (Geladi and Kowalski 1986; Wold et al. 2001). PLS regression was obtained by the software running under Matlab developed by (Nørgaard et al. 2000; Leardi and Nørgaard 2005). Support vector machine (SVM) regression was developed by (Suykens and Vandewalle 1999; Suykens et al. 2002) to deal with non-linear regressions. Computations were obtained with the Matlab toolbox created by the authors (Tange et al. 2015; Ghorbani et al. 2016). Ascending stepwise linear regression (ASLR) was described earlier (Goormaghtigh et al. 2006, 2009). The ascending stepwise linear regression introduces in the model one absorbance value at a time, in an ascending stepwise manner. The result is a linear equation requiring usually only 2–4 absorbance values to obtain a given secondary structure content.

A first validation was obtained by a leave-one-out (LOO) cross-validation. In LOO cross-validation, the evaluation of the error is not based on repeated measurements of the same sample but it uses the full set information to validate the model. One protein at a time was removed from the training set and tested by the model built using the 84 remaining proteins. This was repeated 85 times. The quality of the prediction was evaluated as the root mean square standard error in cross-validation (RMSECV). This error was compared with the standard deviation of the reference (i.e. DSSP values) secondary structure content (STDDEV^REFCV^) by computing ζ^CV^ = STDDEV^REFCV^/RMSECV. While STDDEV^REFCV^ is the error of prediction that would be obtained if guessing that the secondary structure content for each protein is the mean secondary structure in the protein set, ζ^CV^ value reports how much better the model based on spectroscopic data does. It is interesting to note that ζ is inversely related to the square of the correlation coefficient (Fearn 2002). To obtain an independent test set for validation, a single subset of the cSP92 protein spectra was identified using the Kennard-Stone algorithms (Kennard and Stone 1969) that is designed to select a uniform distribution of the secondary structure content. In the present work, 25 proteins were used as test set and 60 for the training set. The error of prediction RMSEKS and ζ^KS^ = STDDEV^REFKS^/RMSEKS were computed as described above.

### Software

Image analysis, spectrum processing and multivariate analyses were all performed with Kinetics, a home-made software running under MatLab (The MathWorks Inc.). The software is freely available for academics upon request.

## Results

**Fig. 1:**
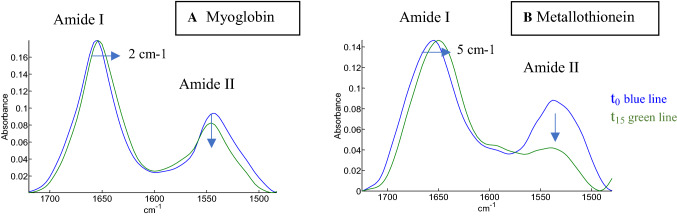
Effect of 15 min deuteration on myoglobin and metallothionein infrared spectra in the amide I—amide II spectral range. Spectra shown at t_0_ (blue lines) and t_15_ (green lines) for myoglobin (**a**) and metallothionein (**b**). An arrow indicates the direction of the changes induced by 15-min deuteration

Infrared images of the microarrays were recorded at 4 time points of the H/D exchange process as described in Material and Methods. A subset of 85 proteins was selected from the cSP92 proteins, after elimination of 7 proteins with poor quality spectra (Table S1). Figure S1 reports the spectra of a highly helical protein spectrum, myoglobin, and a highly disordered protein, metallothionein at t_0_, t_15_, t_105_, t_24h_ between 3600 and 1050 cm^−1^. The main contributions of ^1^H_2_O, ^1^HO^2^H, ^2^H_2_O along with the main protein amide contributions are identified on Figure S1. Figure 1 illustrates the effect of 15-min HDX in the amide I—amide II spectral range for myoglobin and metallothionein. Before exchange (t_0_), the amide I maximum is located at 1655 cm^−1^ for myoglobin as well as for metallothionein, i.e. the contribution of α-helix and disordered structure overlaps to a large extend though the metallothionein band is broader than the myoglobin one (Fig. 1, blue curves). After 15 min deuteration (t_15_), Amide I shifts to 1653 cm^−1^ for myoglobin and 1650 cm^−1^ for metallothionein. Reproducibility is evaluated for the quadruplicates in Figure S2. It must also be stressed that hydration of the film resulting from the addition of ^2^H_2_O in the N_2_ flow flushing the cell could explain a 1–2 cm^−1^ downshift of the amide I (see the discussion section). Concomitantly, a large difference is observed between the t_15_-deuterated protein spectra in the amide II band, indicating a much larger extent of the exchange for metallothionein. HDX causes indeed a reduction of amide II and the simultaneous appearance of amide II' around 1450 cm^−1^ (amide II’ not shown, see Figure S1). It must be mentioned here that the mixed ^2^H-O-^1^H molecule also absorbs at 1450 cm^−1^ (Zuber et al. 1992; Goormaghtigh et al. 1994b). Yet, this contribution is expected to be very small as the sample compartment is flushed with a continuous flow of ^2^H_2_O-saturated N_2_. Any ^1^H_2_O present at the beginning of the experiment would therefore be very rapidly replaced and removed from the cell. The larger extent of exchange in the disordered structure promotes a larger difference in the amide I band position, suggesting that α-helices of myoglobin may be easier to distinguish from disordered structures after a t_15_ HDX period. Such a difference in exchange rates can be assigned to the difference in secondary structure. Intrinsically disordered proteins such as metallothionein (0% H, 0%, E, 100% “Others”) have amide protons more accessible than in well-structured helical protein such as myoglobin (73% H, 0% E, 27% “Others”) where the amide protons are involved in strong H bonds within the helical structures.

**Fig. 2:**
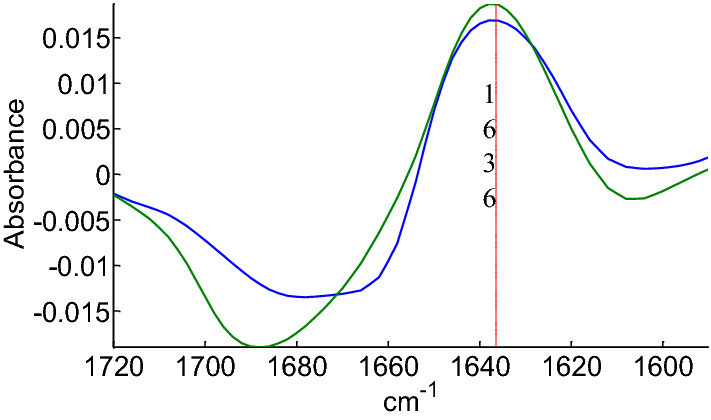
Difference between spectra at t_15_ and t_0_ HDX time for myoglobin (blue) and metallothionein (green). Absorbance at the maximum at 1636 cm^−1^ is indicated

The effect of deuteration at t_15_ is better appreciated after subtracting the t_15_ spectrum from the t_0_ one. The difference spectrum is shown in Fig. 2 for myoglobin (blue) and metallothionein (green). A positive deviation around 1636 cm^−1^ is observed in both cases. This means that the rapidly exchanged amide fraction now overlaps the β-sheet absorption band. This observation suggests it may be more challenging to quantify β-sheet content after 15 min HDX.

**Fig. 3:**
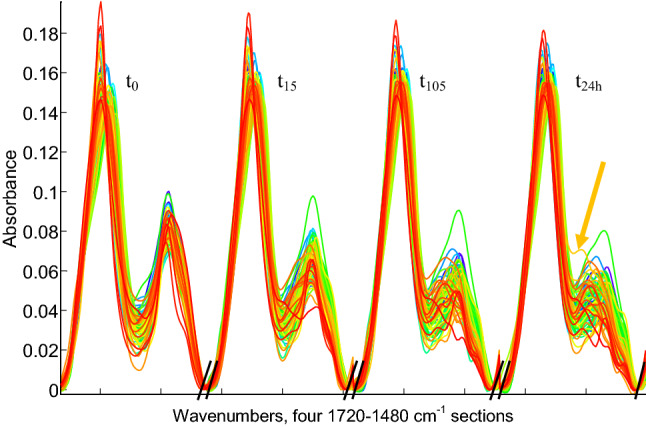
85 concatenate spectra of 4 deuteration exchange period of time, t_0_, t_15_, t_105_ and t_24h_ as indicated on the figure. Spectra were sorted in ascending order of disordered structure content from low (blue) to high (red). Each interval begins at 1720 and ends at 1480 cm^−1^. Limits between intervals are indicated on the abscissa by //. The spectrum of glucagon indicated by the arrow at t_24h_ is discussed in the Discussion section of the paper

As many previous works, mentioned in the introduction, already demonstrated that amide I-amide II region of the protein spectrum (located between 1700 and 1500 cm^−1^) is the most informative regarding protein secondary structure assessment, we restricted the analysis to this spectral range. For the sake of the simplicity of the analysis, as well as for considering potential synergies between different deuteration periods of time, the 1720–1480 cm^−1^ spectral region corresponding to the 4 HDX periods were placed side by side to form a single spectrum for each protein. Concatenate spectra are shown in Fig. 3 for the 85 proteins.

Each combined spectrum now counts 4 regions corresponding to the 4 deuteration periods: t_0_, t_15_, t_105_ and t_24h_ recorded between 1720 and 1480 cm^−1^ (amide I and II bands). An overall decrease in amide II is already visible at the first exchange time t_15_.

As spectra are sorted in Fig. 3 according to their content in disordered structure, it can be observed that the highly disordered proteins (red spectra) display in general a faster drop in amide II intensity at t_15_ than the blue ones. This becomes less clear after 24 h deuteration at t_24h_ as amide II of blue and red spectra tend to overlap. While after 24 h exchange can be far from complete for highly structured proteins (Downer et al. 1986; Vigano et al. 2004), it must be stressed that a very significant part of the absorbance left in the amide II spectral range is due to contributions of amino acid side chains as reviewed in (Goormaghtigh et al. 1994c; Barth 2000, 2007; Wolpert and Hellwig 2006). Depending on side chain composition, this contribution varies but is expected to be around 20% of the amide intensity (Chirgadze et al. 1975; Rahmelow et al. 1998). Deuteration of side chain brings further contributions to the dip located between amide I and amide II bands, for instance, from arginine (Chirgadze et al. 1975), which explains in part the remaining or even increasing intensity observed between amide I and amide II bands.

### Partial least squares PLS

In a first approach, we applied the PLS linear regression method where the predictor variables are the infrared spectra and the dependent variables are the related fractions of secondary structure elements provided by DSSP. The PLS linear regression method was applied on the 85 combined spectra. We first determined the optimal number of latent variables (LVs) specific to each interval corresponding to each deuteration time period and for each structure (α-helices, ordered α-helices, β-sheet, antiparallel β-sheet and “Others”) as in PLS, the calculation is made independently for each structure. The interval PLS method (iPLS) developed by (Nørgaard et al. 2000) was convenient to analyse portions of the spectral range and was aplied here.

**Fig. 4:**
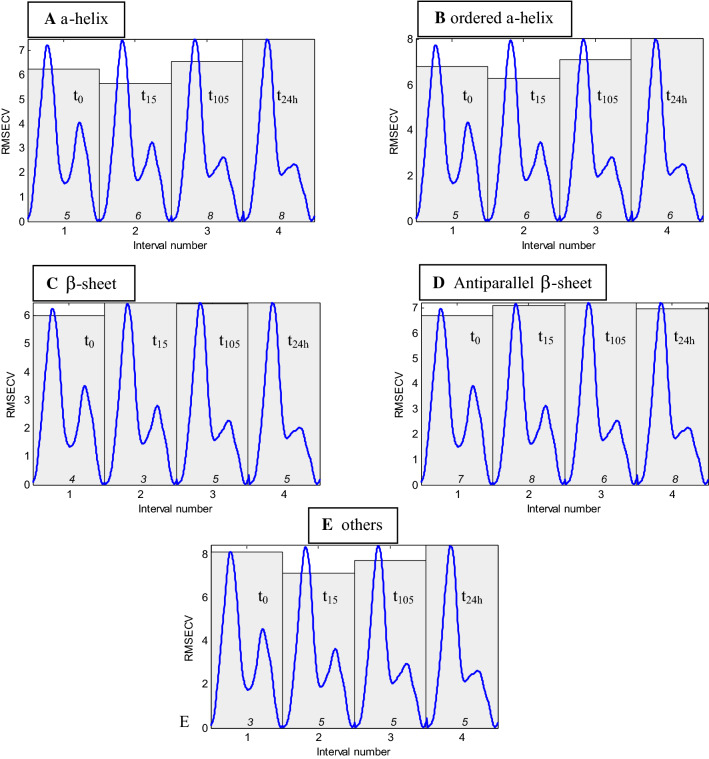
RMSECV profile obtained by iPLS regression on 4 intervals encompassing the 1720–1480 cm^−1^ spectral ranges for 85 protein spectra. Each of the 4 intervals, labelled 1, 2, 3 and 4, includes protein spectra obtained, respectively, at t_0_, t_15_, t_105_ and t_24h_ as indicated on the figure. The RMCECV, calculated in leave-one-out cross validation, are indicated by the shaded area for each of the 4 time intervals. The optimal number of LVs to be used for an optimal prediction is indicated at the bottom of each bin*.* For the sake of the clarity, the mean spectrum at each time point is plotted between 1720 and 1480 cm^−1^ (units not shown) in blue in the corresponding interval. Data are presented for α-helix (**a**), ordered α-helix (**b**), β-sheet (**c**), antiparallel β-sheet (**d**) and “Others” (**e**) content

Figure 4 reports the root mean square error in cross validation (RMSECV) of models obtained for each interval, in the case of α-helix (A), ordered helix (B), β-sheet (C), antiparallel β-sheet (D) and “Others” structures (E). Optimal number of latent variables (LVs) is indicated at the bottom of each interval.

At first glance, major differences immediately appear between α-helix, β-sheet and “Others”. For α-helix and ordered α-helix structures, results are quite similar (Fig. 4a and b), best models (giving the minimal error) are obtained at t_15_. with 6 LVs. Best models are also obtained at t_15_ for “Others” with 5 LVs (Fig. 4e). On the contrary, the minimal RMSECV is obtained at t_0_, in the case of β-sheet with 4 LVs (Fig. 4c) and antiparallel β-sheet with 7 LVs (Fig. 4d).

**Fig. 5:**
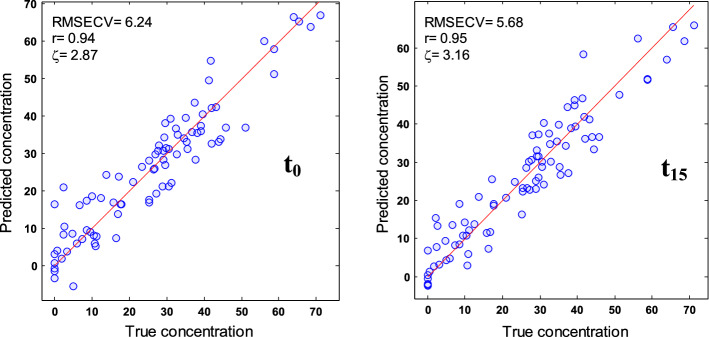
Relation between predicted and actual α-helix content when evaluated at t_0_ (left panel) and t_15_ (right panel). Each circle represents a protein. RMSECV, correlation coefficient *r* and ξ^CV^ are reported in the inset

In conclusion, the most efficient models, i.e. models resulting in the smallest RMSECV, are obtained after partial deuteration at t_15_ for α-helix and “Others” structures. For β-sheets, t_0_ gives better prediction. Further analyses (not shown) indicate that neither smaller subintervals nor the global model that considers the entire spectral range of the concatenate spectra outperform those found at t_0_ or t_15_ (Table 1). Once subintervals giving the minimum error were identified, models for predicting secondary structure content were built using the best deuteration time. Figure 5 reports for the α-helix structure the predicted versus actual content using models built with the 1720–1480 cm^−1^ spectral range recorded at t_0_ and t_15_. Figure 5 indicates a global improvement of the prediction at t_15_ but does not indicate that a specific protein category such as α-helix rich proteins is responsible for the improvement. Similar conclusions were reached for β-sheet and “Others” structures (Figure S3).

**Table 1:** Cross-validation evaluation of secondary structure evaluation at t_0_ and t_15_ and for the global model

	α-helix			Ordered α-helix			Others			β-sheet			Anti//β-sheet		
	RMSECV	n. var	ζ^CV^	RMSECV	n. var	ζ^CV^	RMSECV	n. var	ζ^CV^	RMSECV	n. var	ζ^CV^	RMSECV	n. var	ζ^CV^
PLS t_0_	6.24	5 LVs	2.87	6.8	5 LVs	2.59	8.13	3 LVs	1.2	**6.01**	4 LVs	2.32	**6.72**	7 LVs	2.07
PLS t_15_	**5.68**	6 LVs	3.16	**6.28**	6 LVs	2.8	**7.13**	5 LVs	1.37	6.47	3 LVs	2.15	7.09	8 LVs	1.96
PLS t_GM_	5.95	5 LVs	3.02	6.47	5 LVs	2.72	7.57	4 LVs	1.29	6.23	4 LVs	2.24	7.2	5 LVs	1.93
SVM t_0_	6.17		2.91	6.77		2.6	7.75		1.26	**5.8**		2.4	**6.45**		2.16
SVM t_15_	**6.14**		2.92	**6.65**		2.65	**7.05**		1.39	6.43		2.17	7.09		1.96
SVM t_GM_	6.63		2.71	7		2.51	8.06		1.21	6.42		2.17	6.98		1.99
ASLR t_0_	**5.86**	4 wvnbs	3.06	6.46	4 wvnbs	2.72	7.92	5 wnbs	1.24	5.64	4 wvnbs	2.47	6.54	4 wvnbs	2.13
ASLR t_15_	5.89	4 wvnbs	3.05	6.31	4 wvnbs	2.79	**6.91**	5 wnbs	1.41	5.91	4 wvnbs	2.36	6.76	4 wvnbs	2.06
ASLR t_GM_	5.81	4 wvnbs	3.09	**6.13**	4 wvnbs	2.87	7.21	5 wnbs	1.36	**5.48**	4 wvnbs	2.54	**6.24**	4 wvnbs	2.23
STDDEV^REFCV^	17.94			17.61			9.78			13.93			13.91		

PLS, SVM and ASLR performances in LOO cross-validation in the 1720–1480 cm^−1^ spectral section at t_0_, t_15_ and for the global model (t_GM_) including spectra recorded at t_0_, t_15_, t_105_ and t_24h_. RMSECV, error in prediction (expressed in%), ζ^CV^ = STDDEV^REFCV^/RMSECV. STDDEV^REFCV^ is the standard deviation of the secondary structure content in the protein set. The optimal number of LVs (latent variables) for PLS and wvnbs (wavenumbers) for ASLR are reported. Minimum values are reported in bold

The error of prediction in cross validation, RMSECV, is shown in the inset and is reported for all structures in Table 1. The smaller RMSECV was obtained at t_15_ for α-helix, RMSECV = 5.68%, compared to 6.24% at t_0_. Similarly, the best prediction was obtained at t_15_ in the case of “Others”, RMSECV = 7.13% instead of 8.13% at t_0_. On the contrary, lower RMSECV, 6.01%, was obtained for β-sheet at t_0_ instead of 6.47% at t_15_. When the models were built using a single 25-protein test set selected by the Kennard-Stone algorithm, the finding is essentially the same, confirming with an independent validation test set the conclusions obtained by LOO cross-validation. It must be noted here that for each structure, the 25 proteins of the validation set were selected independently and a calibration model was constructed with the remaining 60 protein spectra. The spectra selected for the validation test are reported in Table S1 for each structure type and results of the validation test are presented in Table 2.

**Table 2:** Evaluation of secondary structure evaluation at t_0_ and t_15_ and for the global model on a 25-protein test set selected by Kennard-Stone algorithm

	α-helix			Ordered α-helix			Others			β-sheet			Anti//β-sheet		
	RMSEKS	n. var	ζ^KS^	RMSEKS	n. var	ζ ^KS^	RMSEKS	n. var	ζ ^KS^	RMSEKS	n. var	ζ ^KS^	RMSEKS	n. var	ζ ^KS^
PLS t_0_	7.13	5 LVs	2.96	7.62	5 LVs	2.73	12.22	3 LVs	1.24	5.36	4 LVs	2.82	6.12	7 LVS	2.48
PLS t_15_	6.63	6 LVs	3.18	7.44	6 LVs	2.8	9.3	5 LVs	1.63	7.07	3 LVs	2.14	6.52	8 LVs	2.33
PLS t_GM_	6.2	5 LVs	3.4	7.22	5 LVs	2.88	10.49	4 LVs	1.45	6.15	4 LVs	2.46	7.41	5 LVs	2.05
SVM t_0_	6.97		3.1	7.21		2.75	10.52		1.37	4.98		3.12	5.66		2.61
SVM t_15_	7.42		2.91	7.3		2.71	9.98		1.44	6.47		2.4	6.62		2.23
SVM t_GM_	7.72		2.8	7.47		2.65	12.42		1.16	6.63		2.35	6.94		2.13
ASLR t_0_	7.49	4 wnbs	2.82	7.9	4 wnbs	2.63	12.54	5 wnbs	1.21	5.25	4 wnbs	2.88	5.89	4 wnbs	2.58
ASLR t_15_	7.07	4 wnbs	2.98	8.07	4 wnbs	2.58	10.31	5 wnbs	1.47	5.66	4 wnbs	2.67	6.2	4 wnbs	2.45
ASLR t_GM_	6.83	4 wnbs	3.09	7.38	4 wnbs	2.82	11.12	5 wnbs	1.37	4.95	4 wnbs	3.06	6.1	4 wnbs	2.49
STDDEV^REFKS^	21.1			20.82			15.18			15.12			15.2		

PLS, SVM and ASLR performances on a 25-protein test set selected by Kennard-Stone algorithm in the 1720–1480 cm^−1^ spectral section at t_0_, t_15_ and for the global model (t_GM_) including spectra recorded at t_0_, t_15_, t_105_ and t_24h_. RMSEKS, error in prediction (expressed in%), ζ^KS^ = STDDEV^REFKS^/RMSEKS. STDDEV^REFKS^ is the standard deviation of the secondary structure content in test set selected by the Kennard-Stone algorithm. The optimal number of LVs (latent variables) for PLS and wvnbs (wavenumbers) for ASLR are reported

### SVM

While PLS modelling is based on multiple linear regressions, on the contrary, support vector machine (SVM) algorithm is designed for non-linear regressions. Results obtained by SVM modelling for all fractions considered are summarized in Table 1 (Figure S4 reports the relation between predicted and actual structure content for α-helix, β-sheet and “Others”). Performances are quite similar at t_15_ and t_0_ for α-helix (RMSECV = 6.14% and 6.17, respectively). “Others” structure is better predicted at t_15_ than at t_0_ (RMSECV = 7.05% and 7.75%, respectively) and β-sheet at t_0_ than at t_15_ (RMSECV = 5.80% and 6.43%, respectively). As for PLS, when the models are built using a single 25-protein test set selected by the Kennard-Stone algorithm, the finding is essentially the same (Table 2), confirming the conclusions obtained by LOO cross-validation on an external validation test set.

### ASLR

**Fig. 6:**
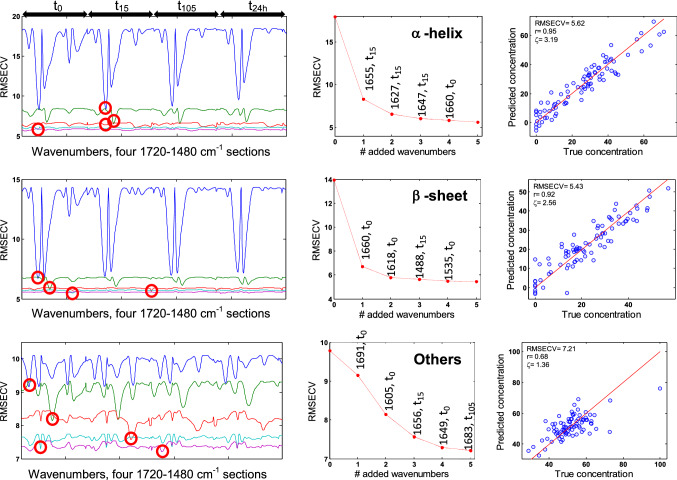
Ascending stepwise linear regression. Top, α-helix; middle, β-sheet; bottom, “Others”. The left column presents the profile of the RMSECV (expressed in % of the structure content) when a single wavenumber is used (blue line), when a second is added (green line), a third one (red line), a fourth one (cyan) and a fifth one (magenta). For each curve, the value of the minimum with the corresponding wavenumber is reported in the middle column. This wavenumber is added to the model and a new scan is started to identify the next best wavenumber that, when added to the previous ones, bring useful information for secondary structure prediction. The right column reports the predicted secondary structure content as a function of the actual content for each structure. The red diagonal line reflects a situation where prediction is perfect. All four deuteration time periods indicated on top of the left columns were used simultaneously. The best wavenumbers identified by a red circle are reported in the middle column.

Ascending stepwise linear regression is a rather simple approach, which introduces step by step wavenumbers in the model to obtain the best multiple regression. The advantage is that the weight of each wavenumber is immediately apparent. Figure 6 (left column) reports the RMSECV profiles along the entire spectral range of the concatenate spectra when a first wavenumber is chosen, then a second is added and so on. The wavenumber providing the smallest root mean square error in cross validation (RMSECV) is retained at each iteration in the algorithmic process, as shown in Fig. 6 (middle column) for α-helix, β-sheet and “Others” (results obtained for ordered α-helix and antiparallel β-sheet are shown in Fig S5). At each wavenumber added, the RMSECV value drops, reflecting the improvement of the model. The RMSECV spectral profile (left column,) allows visualizing the information content provided by each wavenumber. For all structures except “Others”, it reveals that 4 wavenumbers are enough to extract all relevant information necessary to predict the secondary structure. Addition of the fifth one does not bring more information. On the contrary, for “Others”, it appears that 5 wavenumbers are required as there is clearly a level of useful information in the fifth RMSECV profile. This is not surprising in view of the wide variety and complexity of the structures present in this group. Best ASLR prediction models obtained with 5 wavenumbers are shown in Fig. 6 right column.

Table 3 reports the wavenumbers selected in order of relevance for each secondary structure and their assignment to one of the 4 deuteration times. A colour code is applied to each deuteration time (green, t_0_; yellow, t_15_; red, t_105_). It must be noted that t_24h_ is never selected and is therefore not discussed here. Examination of Table 3 reveals that, in the case of α-helix, most information is extracted at t_15_ (t_15_ in yellow), around 1655, 1627 and 1647 cm^−1^. For β-sheet, ordered α-helix, anti//β-sheet and “Others”, the first wavenumbers in order of relevance are located at t_0_. However, some information is drawn from the partially deuterated spectra (t_15_ or t_105_) for wavenumbers used in third and fourth position.

**Table 3:**
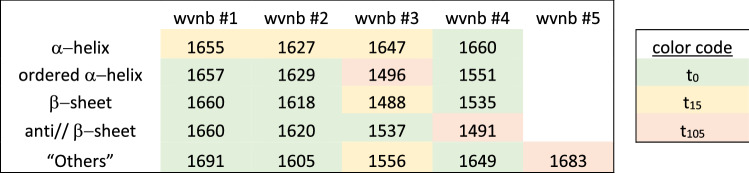
Wavenumbers selected by order of relevance for α-helix, ordered α-helix, β-sheet, antiparallel β-sheet and “Others” and their deuteration time. A colour code identifies each period: t0, green; t15, yellow and t105, red

Results were obtained for the entire concatenate spectra. Models obtained for each individual deuteration time are summarized in Table 1

Unlike the results obtained by PLS and SVM, the best ASLR predictions are obtained when working on concatenate spectra (RMSECV = 5.81% for α-helix, 6.13% ordered α-helix, 5.48% β-sheet, 6.24% antiparallel β-sheet), except for “Others” structure for which RMSECV is better at t_15_ (RMSECV = 6.91%, Table 1).

Once again, when the models are built using a single 25-protein test set selected by the Kennard-Stone algorithm, the finding is essentially the same (Table 2), confirming the conclusions obtained by LOO cross-validation on an external validation test set.

## Discussion

**Fig. 7:**
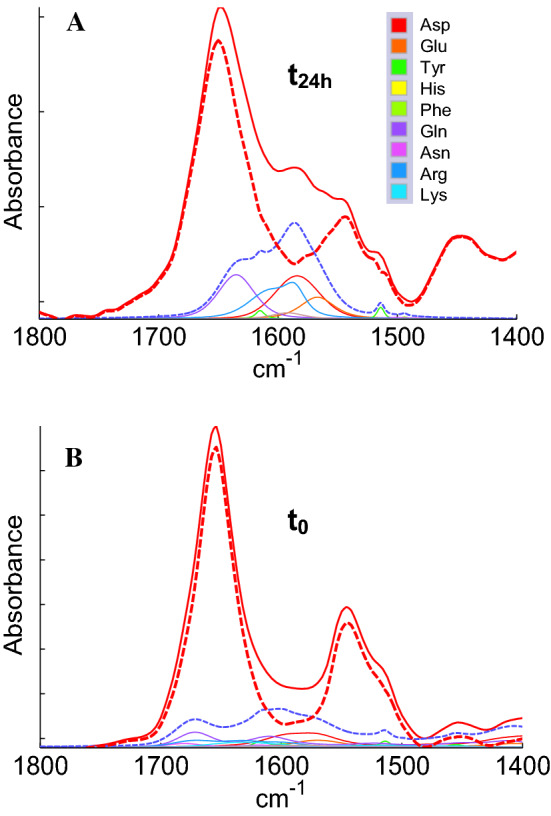
Glucagon spectrum red plain line in the deuterated (t_24h_, panel A) and protonated (t_0_, panel B) forms. The contribution of the different side chains (see legend in panel A.) and their sum (blue dashed line) are presented in both cases. After subtraction of the side chain contributions, the corrected spectra appear as dashed red lines

Table 1 provides an overview of the results obtained by PLS, SVM and ASLR. RMSECV values are reported for all structures. When comparing the different deuteration times, best estimation by PLS and SVM are obtained at t_15_ for α-helix (RMSECV = 5.68–6.14%, respectively) and for “Others” (RMSECV = 7.13 and 7.05%). %). In no case was the full model using the entire concatenated spectra better than t_15_ taken alone. As far as the β-sheet fraction is concerned, it is on the contrary at time t_0_ that the modelling is more performant by both PLS and SVM (RMSECV = 6.47% and 5.8). Tables 1 and 2 indicate a relative improvement of α-helix and “Others” prediction and a relative degradation of the β-sheet structure content prediction after a short (15 min) deuteration period. The rationale is that the “Others” structure exchange must faster than the α-helix. The “Others” IR contribution in the amide I shifts therefore more rapidly towards lower wavenumbers. As already illustrated in Fig. 1, after 15 min, disordered structures, dominant in the “Others” class, have been mostly exchanged while the α-helix structure is not. The consequence is that the overlap of the two contributions is transiently decreased, resulting in an improvement of the predictions for these two structures. As the β-sheet structure exchange slowly, after 15 min deuteration, the deuterated contribution of the random structure overlaps the high wavenumber contribution of the β-sheet, de facto decreasing the quality of the β-sheet content prediction. Another aspect can explain the relative degradation of β-sheet content prediction after deuteration. Side chain absorptions present in the amide I amide II range of the spectrum also shift and overlap the β-sheet spectral region. This is particularly significant when the protein is rich in arginine, glutamine, asparagine and lysine. Careful examination of Fig. 3 reveals an orange spectrum, which is obviously more intense near 1600 cm^−1^ at t_24h_ than at t_0_. It is the spectrum of glucagon (indicated by an arrow in Fig. 3) which has an arginine content of 6.9% and a glutamine content of 10.34% instead of a mean value of 4.3% and 3.7%, respectively, in cSP92 protein set. In fact, no other protein has as much glutamine as glucagon in cSP92, the second richest one is ubiquitin with 7.89%. The main absorption band of glutamine is found at 1672 cm^−1^ in the protonated form (Venyaminov and Kalnin 1991; Wolpert and Hellwig 2006) and 1635 cm^−1^ in the deuterated form (Chirgadze et al. 1975), i.e. exactly where the β-sheet structure absorbs. The effect of amino acid side chain deuteration is illustrated in Fig. 7. In Fig. 7, glucagon spectrum is represented by the red plain line in the protonated (panel B) and deuterated (panel A) states along with the contribution of the different side chains and their sum (blue line). It is clear that deuteration results in an enhanced absorbance in the 1620–1580 cm^−1^ spectral range that can interfere with the evaluation of the β-sheet structure content. After subtraction of the sum of the amino acid side chain contributions, glucagon corrected spectra are presented as dashed lines. The effect is striking. Yet, even though correction for side chain contributions has been attempted (Goormaghtigh et al. 1996; Raussens et al. 2004; Goormaghtigh 2009) and parameters describing side chain band shapes have been reviewed elsewhere (Goormaghtigh et al. 1994c; Barth 2000, 2007), the success of the process is limited by the current impossibility to describe correctly the wealth of variations that exist in these contributions. Yet, Fig. 7 indicates that for glucagon, an improvement is obtained as after correction, the corrected spectrum resembles the usual spectrum of a partially deuterated protein.

The best ASLR models were obtained when considering the entire concatenate spectra instead of t_0_ or t_15_ period alone. This can be understood as ASLR picks up the best wavenumbers one by one and is not disturbed by the rest of the spectrum. Intriguingly, limiting ASLR to one deuteration time (t_15_) improves the prediction for the “Others” structure with respect to the full length concatenated spectra. It must be stressed here that ASLR selects the first best wavenumber, which remains unquestioned when searching for the second one. This one-way path may select a path that is not optimal for the prediction, as exemplified by the observation that restricting the data to t_15_ finally ends up with a better model.

As a preliminary to further discussion, a comment is needed about the measure of the quality of secondary structure evaluations. Two informative quantities regarding the quality of the prediction have been used, the error of prediction (RMSECV) on the one hand and ζ^CV^ defined as the ratio of standard deviation of reference values (STDDEV^REFCV^) over RMSECV on the other hand. While the former inform on the absolute error, the latter is a measure of prediction efficiency. Importantly, as the latter refers to STDDEV^REFCV^, the ζ^CV^ figures depend on STDDEV^REFCV^. Since the standard deviation of the reference data depends on the dataset and is therefore different for each structure and each test set, the ζ value should therefore be used with caution when comparing ζ for different structures or different protein test sets. It can be understood that for similar RMSEs (RMSECV and RMSEKS), the ζ values are generally higher for the 25-protein test set which has in general higher STDDEV^REFKS^ because spectra are selected, by design, to span homogenously the concentration range available. Obviously, this does not mean the models built with 60 proteins are better than the RMSECV models built using the 85 proteins but it largely reflects the breadth of structure sampling in the test set. The α-helix fraction is predicted at t_15_ with an error RMSEKS of 6.63% by PLS (Table 2) and RMSECV 5.68% (Table 1) in cross-validation. Yet, the ζ^KS^ and ζ^CV^ scores are, respectively, 3.18 and 3.16 reflecting the fact that, even if RMSECV is higher than for the 25-protein set, the apparently better RMSE is due to a larger STDDEV^REFKS^ in the 25 proteins selected by Kennard-Stone algorithm than in the full 85 protein set (21.1 and 17.9%, respectively). However, it is important to note that the prediction remains good for a 25-protein independent set. For β-sheets, the prediction is very good (RMSEKS around 5.0%) at t0. The relatively poor prediction for the “Others” fraction (RMSEKS: 9.3% in the best case) can be explained by the removal of the metallothionein from the calibration set (Table S1). Metallothionein is the sole intrinsically disordered protein in the 85 protein. This limitation is due to the fact that finding disordered proteins available with a high-resolution structure deposited in the PDB is indeed challenging. Because metallothionein is rather unique, to be correctly predicted, metallothionein must be part of the calibration set. In this case, the RMSEKS for “Others” will drops from 9.3 to 7.23% (not shown). The global overview presented in Tables 1 and 2 also indicates that the ordered helices are not better predicted than α-helices (i.e. ordered and disordered helices taken together) at the opposite to previous observations reported in the literature (see "Introduction"). Similarly, the antiparallel β-sheet is not better predicted than the β-sheet (i.e. the antiparallel β-sheet and parallel β-sheet taken together).

The hydration level of the sample before deuteration starts could be a concern for protein structure stability and for FTIR spectra. Early works by Prestrelski et al. (Prestrelski et al. 1993; Carpenter et al. 1993) described that most proteins do not experience structural changes by lyophilization. It must be stressed that the samples used in this work are less desiccated than lyophilized samples. Protein films contain indeed a fair amount of water. It was determined by ^2^H NMR combined with FTIR measurements that the lowest amount of water that can be reached upon exposing protein films to dry air was 0.13 g water per g protein (de Jongh et al. 1996). In uncontrolled conditions (open air), the amount of water was always above 0.25 g water per g protein. At that concentration, the water spectrum does not vary anymore with the water/protein ratio, the protein carboxylic and carbonyl sites are saturates (Careri et al. 1980) and enzyme activity can be measured for lysozyme (Careri et al. 1980). Poole and Finney (Poole and Finney 1984) reported the sequential hydration of lysozyme and α-lactalbumin. For both proteins, most of the hydration effects occur below 0.13 g water per g protein. The rate of exchange was also found to reach a maximum above 0.15 g water per g protein (Schinkel et al. 1985). Altogether, these observations reported in the literature suggest that the effect of low hydration at the beginning of the experiments described here is negligible.

Hydrogen deuterium exchange (HDX) has long been used for protein structure and dynamics analysis by FTIR spectroscopy (Zhang et al. 1992; de-Jongh et al. 1995; de Jongh et al. 1997; Scheirlinckx et al. 2004), Raman (Hildebrandt et al. 1993), NMR (Wagner 1983; Zhang et al. 1995) and mass spectroscopy (Nabedryk-Viala et al. 1976; Engen and Smith 2000). Exchangeable hydrogens are distributed all along the main-chain and side chains of proteins. They participate to a dynamic process of exchange with hydrogen atoms of the solvent (Englander et al. 1996). They are also involved in hydrogens bonds that stabilize α-helices and β-sheets. Solvent and protein protons exchange occurs naturally at variable exchange rates. The hydrogens of the peptide groups (1 hydrogen per amino acid except proline) exchange at variable rates depending on their accessibility to solvent and involvement in H-bonds. They have therefore been used as sensors of the conformational state of the protein (Zhang et al. 1992, 1995; Goormaghtigh et al. 1994d, 2009; Englander et al. 1996; Raussens et al. 1996, 2004; Meskers et al. 1999; Scheirlinckx et al. 2004). In principle, hydrogens present in disordered structures are rapidly exchanged with deuterium when compared with protons involved in secondary structure stabilization or buried in hydrophobic clusters (Englander and Kallenbach 1983b; Englander et al. 1996; Skinner et al. 2012a; Englander and Mayne 2014). Yet, the static and dynamic determinants of the exchange remain unclear. Indeed, unexpected slow hydrogen exchange may be observed on the surface of proteins as well as fast exchange of buried hydrogen which may be related to mechanisms of transitional and localized unfolding (Englander and Kallenbach 1983b; Skinner et al. 2012b, a; Englander and Mayne 2014). The best performance obtained for α-helix at the partial deuteration time period t_15_ could be explained by the displacement towards lower wavenumbers (Fig. 2) of the protein fractions that exchange very quickly, likely composed of more accessible structures such as the unordered (random) fraction. As α-helix and random absorbance bands overlap widely, their boundaries before deuteration are blurred and a separation induced by the partial H/D exchange leads to a better analysis of both the helices and “Others” contributions as observed in this paper. As these shifted contributions move towards lower wavenumbers, they overlap β-sheet contributions. This, with a further contribution of amino acid side chains, probably explains the observed degradation of β-sheet evaluation at t_15_ with respect to t_0_. It is also interesting to note that earlier determination of protein secondary structures from FTIR spectra were carried out on deuterated proteins (Byler and Susi 1986; Goormaghtigh et al. 1989, 1990), essentially to avoid the strong overlap between water O–H bending and amide I (Pastrana-Rios 2001). The present work indicates that long deuteration time are not the best choice for secondary structure evaluation.

The present work confirms on a large set of proteins that neither are the ordered/disordered helices quantified better than the full α-helix nor is the split of β-sheet into parallel and antiparallel adequate even though for some specific cases such as amyloid-forming proteins, it appears to be quite relevant (Cerf et al. 2009; Celej et al. 2012). In the present case, it is possible that the mix of sheets of various lengths and made out of various number of strands blurred the difference that is obvious in amyloid β peptides.

Taken together, the results obtained in this work indicate that using deuteration do not bring a breakthrough in secondary structure prediction. There are several reasons why there is a limit to secondary structure prediction accuracy. The major one is related to the simplification of the secondary structure definition. The three classes, α-helix, β-sheet and “Others” are far to form homogenous entities. While this is obvious for the “Others” that, by definition, groups a series of diverse structures described in the introduction, the α-helix category for instance, also contains a wide variety of structural characteristics that yield different FTIR features (variation in band position and bandwidth). Some helices are long, other short, some are bended, some include kinks etc. All these structural characteristics affect the FTIR spectrum. There is therefore not one α-helix spectrum but a wide variety of them centred on the “typical” α-helix spectrum. The definition itself of the α-helix structure relies on rather arbitrary cutoff for hydrogen bond energy or length/angle and backbone φ/ψ angles. From a same high-resolution structure, we decided here to use DSSP designed by Kabsch and Sander (Kabsch and Sander 1983) but several other definitions have been proposed such as STRIDE (Frishman and Argos 1995), XTLSSTR (King and Johnson 1999), KAKSI (Martin et al. 2005), PALSSE (Majumdar et al. 2005) and STICK (Taylor 2001). These methods result, on the average, in 20% difference in the α-helix content but the difference can be much larger for some proteins. In a previous work (De Meutter and Goormaghtigh 2020a), we showed that DSSP is one of the definitions that has the best match with the FTIR spectra. Yet, the different definitions highlight the difficulty to summarize the variations that exist in protein structure in a single category such as α-helix. In addition to definition issues, the variance of a defined structure content may be insufficient to build a prediction model. For instance, the “Others” content in the present database has a mean value of 51.7% but a rather small standard deviation (9.8%). Band overlap is another issue. The present work indicates that replacement of the amide proton by a deuteron is not sufficient to fully solve the problem. Another problem comes from side chain absorption in the amide I—amide II spectral region. This issue has been raised in this paper, e.g. in Fig. 7. The means to bring a perfect correction to side chain contributions are still lacking. Finally, the reference protein set used to calibrate the prediction is critical. The cSP92 protein set, with very well characterized protein sequence, purity and structure quality (De Meutter and Goormaghtigh 2020b) has been designed for this purpose. Yet, it must be emphasized that it contains only protein for which a high-resolution structure is available. The large class of intrinsically disordered proteins is an example of proteins for which it is difficult to obtain a high-resolution structure, as these proteins usually do not crystallize. There is therefore an obvious lack of representation of these proteins with respect to their expected natural abundance (Ward et al. 2004; Weathers et al. 2006). Finally, it must be noted that, in general, the high-resolution structures have been obtained on protein crystals where the protein structure may be slightly different from the conformation present in aqueous solution or in dry film. However, systematic comparison of X-ray and NMR-derived structures indicate that both are very similar when the fluctuations inherent to NMR are taken into account, lending support to the validity of both methods to describe native in vivo structures (Faraggi et al. 2018). Regarding the comparison between FTIR data obtained for protein solution and “dry” film, it must be stressed that, as discussed above, the amount of water left in “dry” proteins is quite significant and the validity of “dry” film has been reviewed elsewhere (Goormaghtigh et al. 1999). FTIR spectra of proteins in the “dry” and solution state show definite differences but the quality of secondary structure prediction is identical, at least for the protein library tested (Goormaghtigh et al. 2009b). All together, the different limitations identified above suggest that we are reaching the limit of the accuracy of structure prediction from FTIR spectra of proteins.

## Supplementary Information

Below is the link to the electronic supplementary material.

Supplementary file1 (PDF 928 KB)

## Data Availability

Materials will be available on request from the authors.

## Code availability

No software was written for this work.
