# Supplementary material for: Evaluation of protein secondary structure from FTIR spectra improved after partial deuteration
Source: Eur Biophys J. 2021 Feb 3;50(3-4):613–28. doi: 10.1007/s00249-021-01502-y (PMC8189984; doi:10.1007/s00249-021-01502-y)
Supplement: Supplementary file 1 — Supplementary file1 (PDF 928 KB) [file 249_2021_1502_MOESM1_ESM.pdf]

Supplementary materials for

Evaluation of protein secondary structure from FTIR spectra improved after partial deuteration

Joëlle De Meutter and Erik Goormaghtigh

Center for Structural Biology and Bioinformatics, Laboratory for the Structure and Function of Biological Membranes, Campus Plaine CP206/02; Université Libre de Bruxelles, B1050 Brussels, Belgium

Table of Contents

Table S1: list of 25 proteins included in the Kennard-Stone test set S2
Figure S1: HDX spectra of myoglobin and metallothionein S4
Figure S2: Reproducibility: quadruplicate spectra of myoglobin and metallothionein at t_0 and t_{15} S5
Figure S3: predicted versus actual secondary structure contents obtained by PLS S6
Figure S4: predicted versus actual secondary structure contents obtained by SVM S7
Figure S5: ASLR determination of secondary structures S8

Table S1: list of 25 proteins included in the Kennard-Stone test set

		Kennart-Stone selection				
Protein name	PDB name	a-helix	ordered a-helix	b-sheet	anti// b-sheet	Others
Aldolase A	1ZAH					
Alpha-2-Macroglobulin	4ACQ		X	X	X	
Alpha-Crystallin B chain	2YGD			X		
Amidase	2UXY			X		
Amino acid oxidase, D-	1VE9					
Apolipoprotein E3	1H7I	X	X			X
Apo-Transferrin	4H0W					
Aprotinin (Trypsin inhibitor Kunitz type)	4Y0Y	X	X	X		X
Avidin	1VYO				X	
Beta-Amylase	1FA2				X	
Beta-Galactosidase	5A1A			X	X	
Beta-Glucunoridase	3LPF					
Beta-Lactamase TEM	1XPB	X			X	
Beta-Lactoglobulin	3NPO	X		X		
Bowman-Birk proteinase inhibitor	5J4Q					X
Calmodulin	1PRW	X	X	X		
Carbonic anhydrase 1	1HCB				X	
Carbonic anhydrase 2	1V9E	X	X			X
Carboxyl esterase	1K4Y					
Carboxypeptidase A1	2CTB			X		
Carboxypeptidase Y	1YSC					
Catalase	3RGP		X			
Ceruloplsamin	4ENZ					
Choline oxidase	4MJW	X				
Chymotripsinogen A	2CGA				X	
Citrate synthase	3ENJ	X				X
Concanavalin A (Lectin)	1I3H				X	X
Creatine (phospho)kinase	1U6R					
Cytochrome c	1HRC					
Deoxyribonuclease-1	3DNI				X	X
DT-diaphorase	1D4A					X
Elastase	1QNJ			X		
Endo-1,4-beta-xylanase	2JIC	X		X	X	
Enolase	1EBH					
Galactose oxidase	2EIE			X	X	
Gelonin	3KTZ	X			X	
Glucagon	1NAU		X			X
Glucose Oxidase	1CF3	X				X
Glutamate oxaloacetate transaminase 1	5TOQ	X				
Glutathione Reductase	3DJG				X	
Glyceraldehyde-3-phosphate dehydrogenase	1J0X			X		
Glycogen phosphorylase-b	1AXR					X
Hemoglobin	2QSP					X
Hexokinase	1IG8					X
Immunoglobuling G	1HZH	X		X	X	X
Insulin (neat)	3W7Y		X		X	
Lactate Dehydrogenase	3H3F					
Lactoferrin (Lactotransferrin) human	1CB6			X		X

Lactoferrin bovin	1BLF		X			
Lactoperoxidase	6A4Y			X		
Lectin	1LEN			X		
Leptin	1AX8	X	X			X
Lipoxidase	1F8N					
Lysostaphin	4LXC			X	X	X
Lysozyme	4LZT		X			
Metallothionein-2A	4MT2					X
Micrococcal Nuclease	1EY0	X				
Myoglobin	1WLA	X	X			X
Ovalbumin	1OVA			X	X	X
Ovotransferrin (Conalbumin)	1OVT			X		
Pepsin A	4PEP		X			
Pepsinogen	2PSG	X				
Peroxidase	1HCH		X			
Phosphatase, Alkaline	1Y6V				X	
Phosphoglucomutase 1	5EPC				X	
Phosphoglycerate kinase	1QPG	X				
Phospholipase A2	2OSH	X	X			
Protein disulfide isomerase	4EL1	X	X		X	
Pyrophosphatase inorganic	1I40	X				X
Pyruvate Kinase	1A49		X			
Ribonuclease A	1KF5		X	X		
Ribonuclease T1	1RLS					
Serum Albumin	1N5U	X		X	X	X
SilB-C	2L55				X	
Silb-NM2	5A4G				X	
Subtilisin Calsberg	3UNX	X			X	
Superoxide Dismutase (Fe)	1ISA	X	X	X		
Superoxide Dismutase (CU Zn)	1Q0E		X			X
Thaumatin	3AOK		X	X		
Transketolase	2R8O			X		
Transthyretin (Préalbumin)	1TTA		X	X		X
Triose Phosphate Isomerase	1YPI		X			
Trypsin inhibitor A Kunitz type SBTI	1BA7	X	X		X	X
Ubiquitin	2WWZ					
ZneB	3LNN		X			

Table S1: alphabetically sorted list of the 85 proteins included in the study along with the selection by the Kennard-Stone algorithm of a 25-protein test set for α-helix, ordered helices, β-sheet, antiparallel β-sheet and "Others" Further information can be found in (De Meutter and Goormaghtigh, 2020) including source organism, expression system if any, commercial reference of the product, Swiss-Prot entry, UniProtKB accession number, PDB entry, resolution of the structure, molecular weight, isoelectric pH, extinction coefficient at 280 nm, content, all secondary structure contents and all 20 amino acid content.

Reference

De Meutter J, Goormaghtigh E (2020) A convenient protein library for spectroscopic calibrations. Comput Struct Biotechnol J 18:1864–1876. https://doi.org/10.1016/j.csbj.2020.07.001

Figure S1: HDX spectra of myoglobin and metallothionein

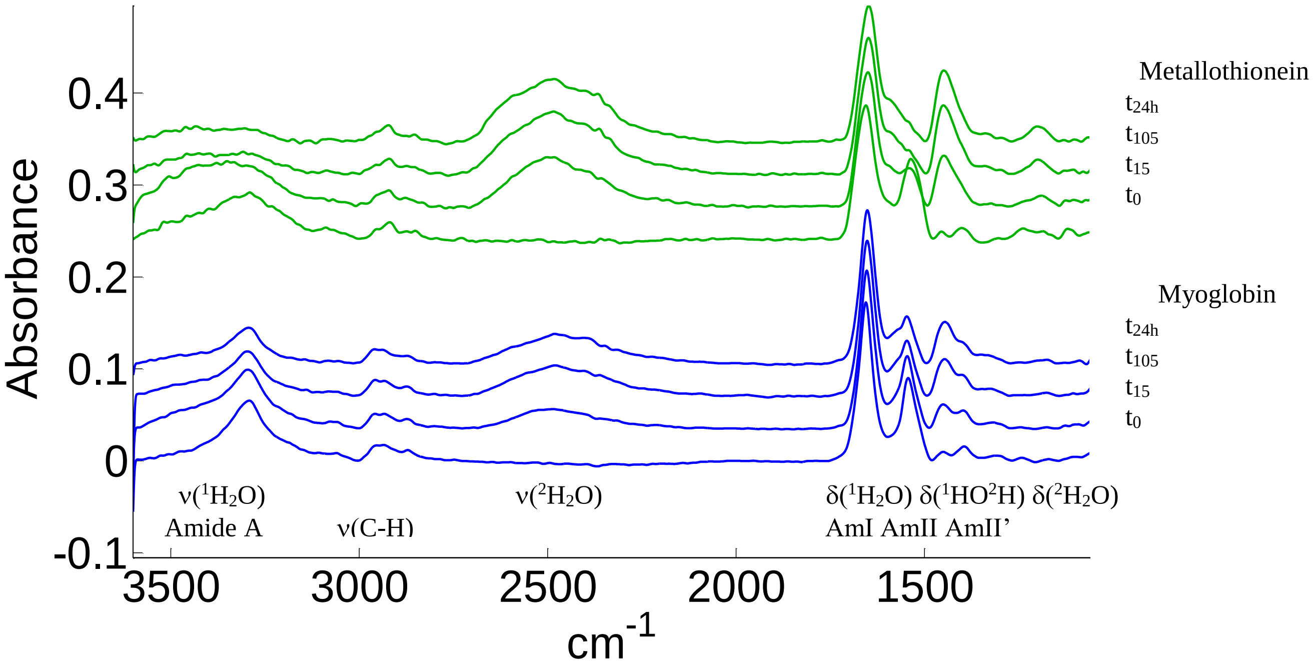

Figure S1: Spectra of myoglobin (blue) and metallothionein (green) recorded at t_0, t_{15}, t_{105}, t_{24h} as indicated on the figure. ν(^1H_2O) at 3300-3500 cm^{-1} refers to ν(O–^{1}H) in water, overlapped by the protein amide A band, ν(^2H_2O) at 2500 cm^{-1}refers to ν(O–^{2}H) in heavy water, δ(^1H_2O) near 1640 cm^{-1} refers to δ(O-^{1}H) in water, δ(^{1}HO^{2}H) near 1450 cm^{-1} to bending in the mixed molecule ^{1}HO^{2}H and δ(^2H_2O) near 1200 cm^{-1} to δ(O-^{2}H) in heavy water. Amide I occurs near 1650 cm^{-1}, amide II near 1545 cm^{-1} and amide II' near 1450 cm^{-1}.

Figure S2: Reproducibility: quadruplicate spectra of myoglobin and metallothionein at t_0 and t_{15}

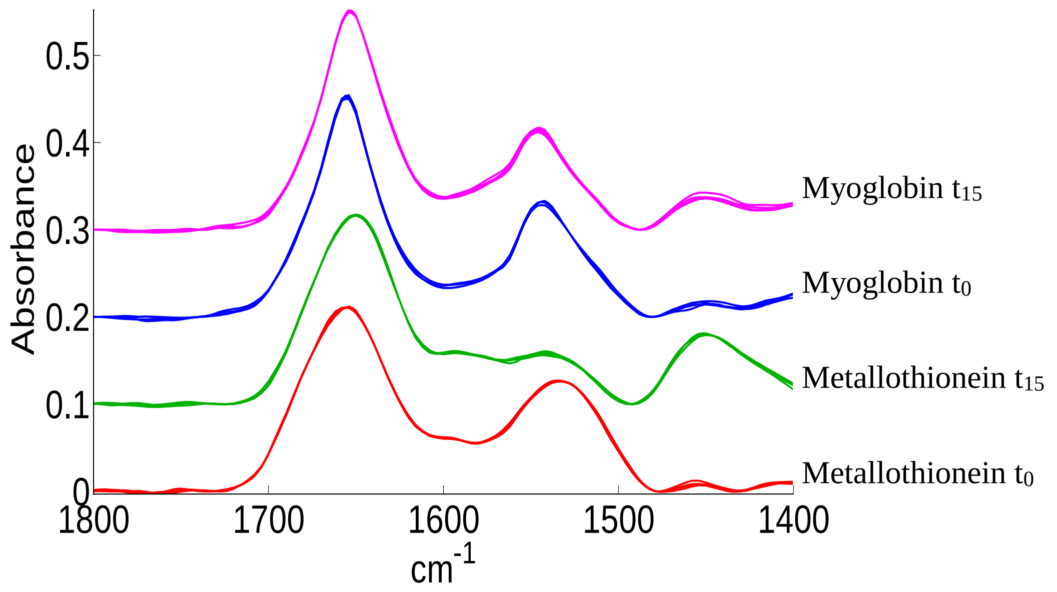

Figure S2: myoglobin and metallothionein spectra are presented at t_0 and t_{15} as indicated in the right margin of the figure. Each condition, represented by a different color, is reproduced in quadruplicate. For the sake of the clarity, each group of 4 spectra has been shifted by 0.1 absorbance unit with respect to the previous one. Spectra have been normalized on the amide I region by vector normalization.

A accurate evaluation of the position of the maximum is obtained after curve fitting of the maximum of the amide I band by a 3rd order polynomial. The mean position of the amide I maximum is presented below with the standard deviation.

**Condition**	**Mean ± standard deviation**
metallothionein t_0	1655.2±0.8
metallothionein t_{15}	1650.1±0.8
myoglobin t_0	1655.5±0.3
myoglobin t_{15}	1653.2±0.1

Position of the amide I maximum ± the standard deviation for the quadruplicates

Figure S3: predicted versus actual secondary structure contents obtained by PLS

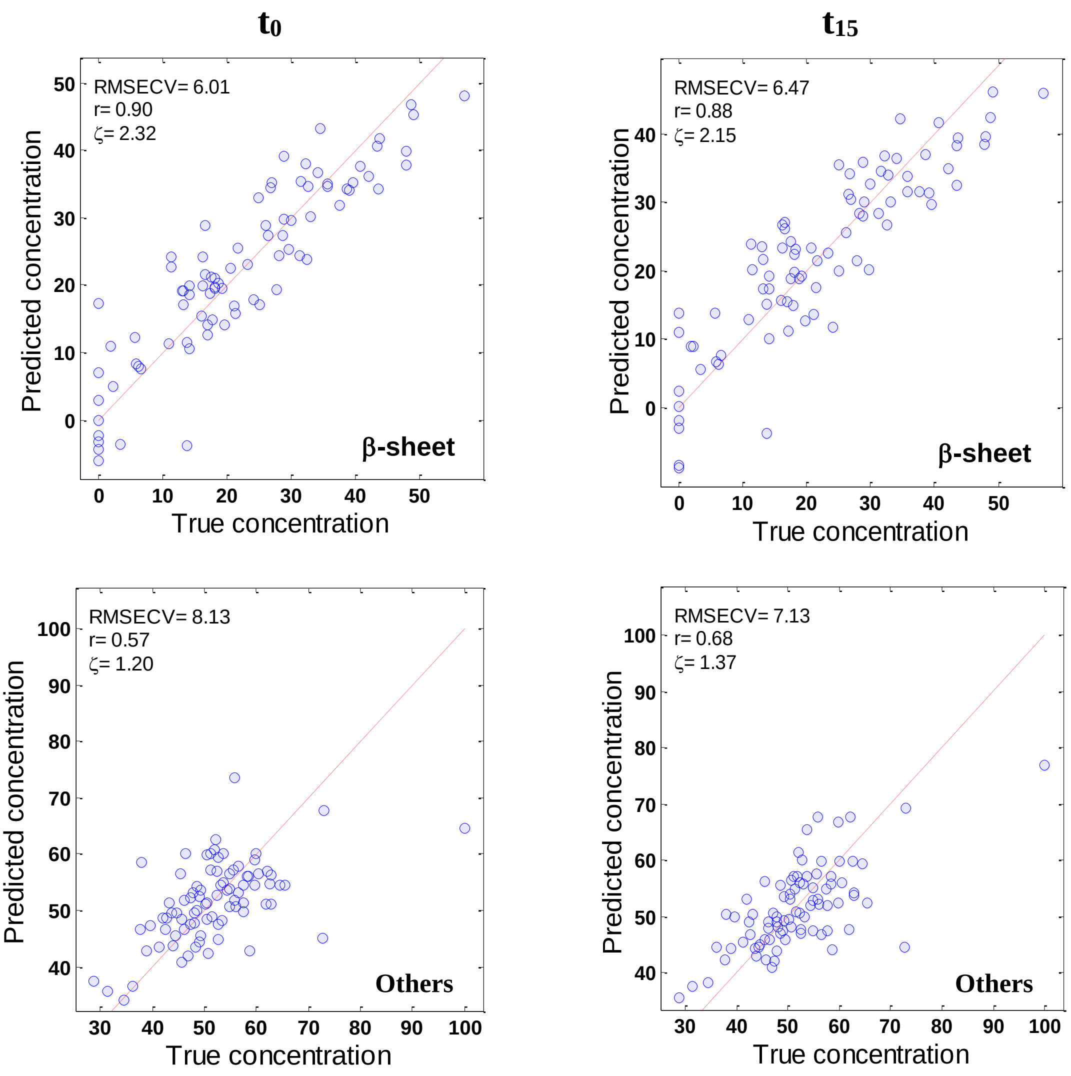

Figure S3: Relation between predicted β-sheet (top row) and "Others" (bottom row) structures obtained by PLS and actual "true" secondary structure content obtained after analysis of PDB-stored high resolution structures by DSSP. All concentrations are reported in %. The left column refers to results obtained at deuteration time t_0 and the right column at t_{15}. Each circle in the graphs represents a protein. RMSECV, correlation coefficient r and ζ^{RMSECV} are reported in the inset.

Figure S4: predicted versus actual secondary structure contents obtained by SVM

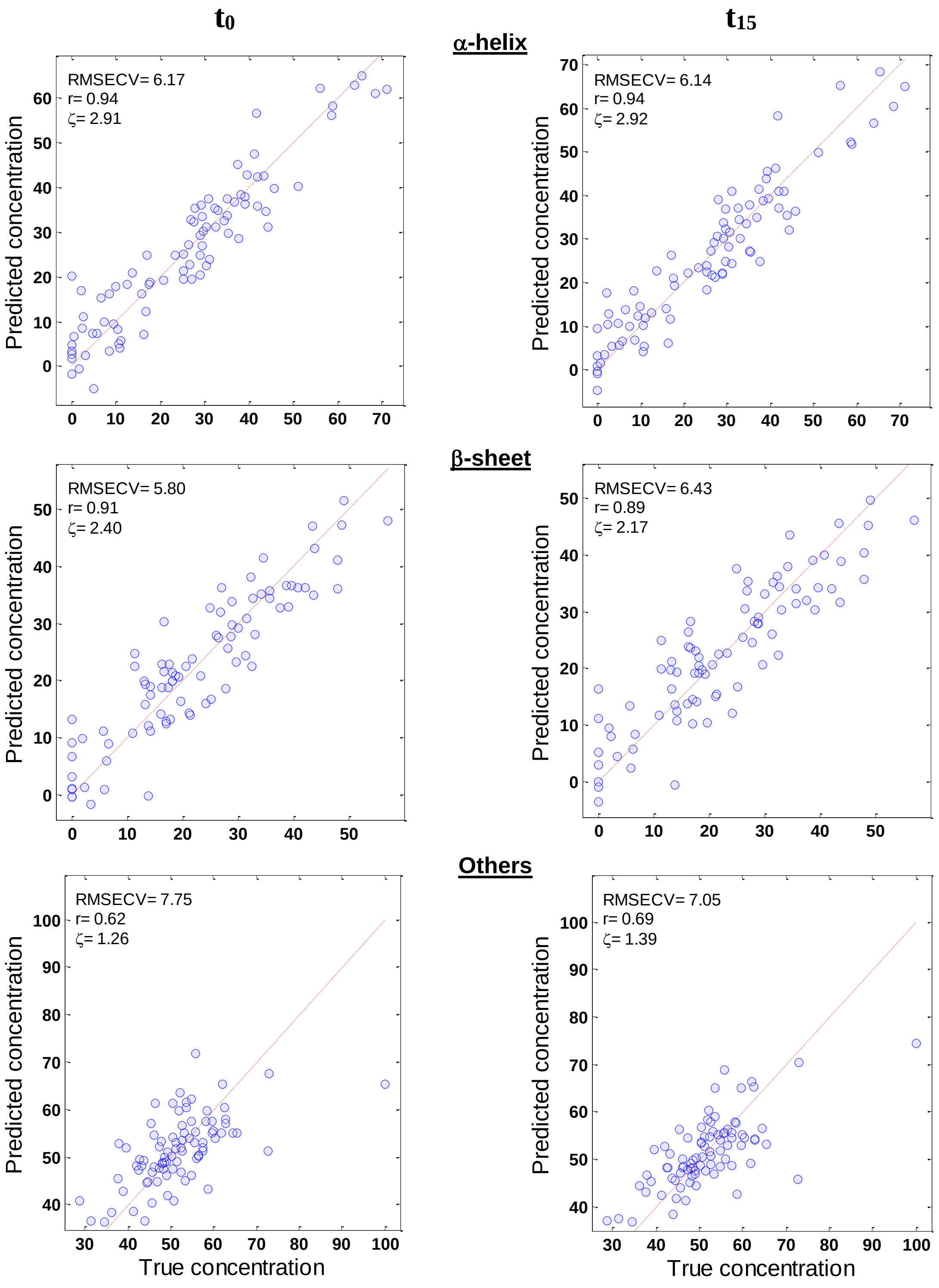

Figure S4: Relation between predicted α-helix (top row), β-sheet(middle) and "Others" (bottom row) structures obtained by SVM and actual ("True") secondary structure content obtained after analysis of PDB-stored high resolution structures by DSSP. All concentrations are reported in %. The left column refers to results obtained at deuteration time t_0 and the right column at t_{15}. Each circle in the graphs represents a protein. RMSECV, correlation coefficient r and ζ are reported in the inset.

Figure S5: ASLR determination of secondary structures

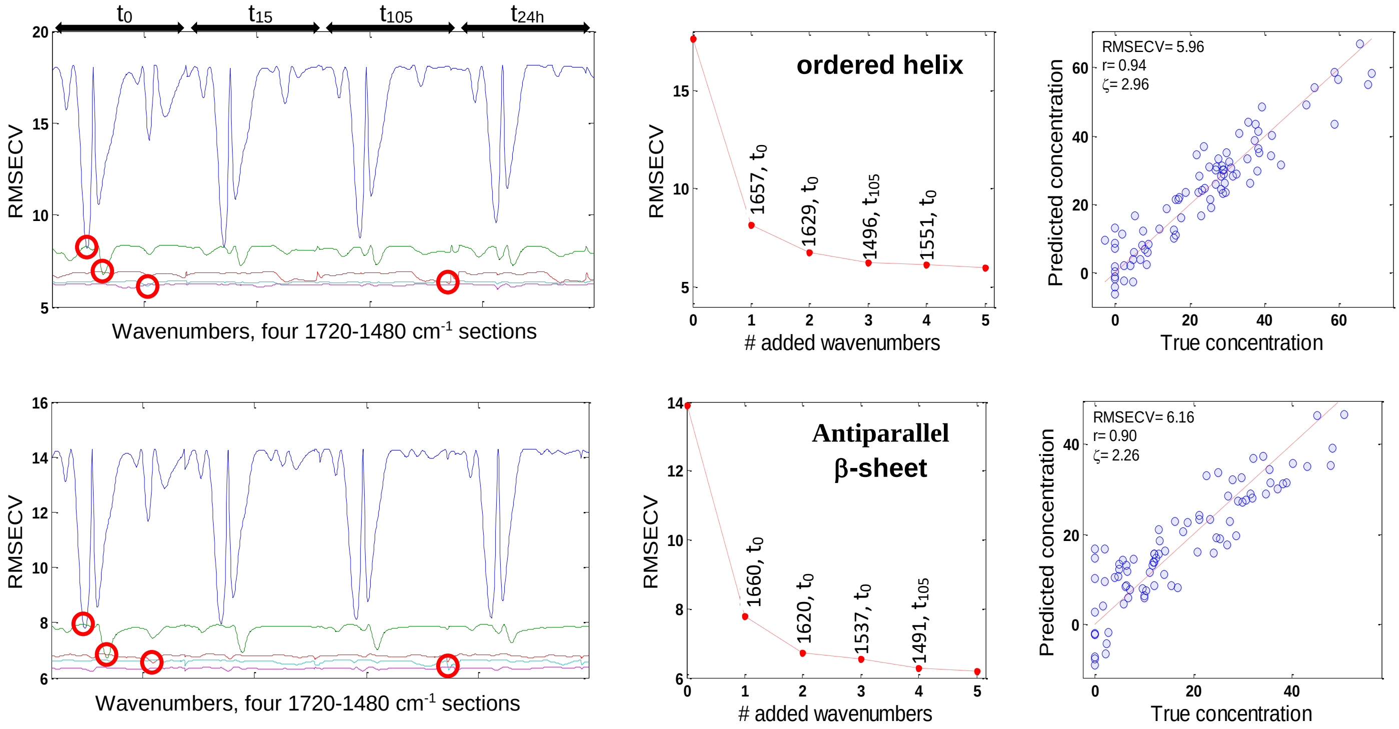

Figure S5: ascending stepwise linear regression. Top, ordered helix; bottom, antiparallel β–sheet. The left column presents the profile of the RMSECV (expressed in % of the structure content) when a single wavenumber is used (blue line), when a second is added (green line), a third one (red line), a fourth one (cyan) and a fifth one (magenta). For each curve, the value of the minimum with the corresponding wavenumber is reported in the middle column. The right column reports the predicted secondary structure content as a function of the actual content for each structure. The red diagonal line reflects a situation where prediction is perfect. All four deuteration time periods indicated on top of the left columns were used simultaneously. The best wavenumbers identified by a red circle are reported in the middle column.
